# A missing link in the METTL3 chain: Chaperone regulation of METTL3 as a BAG:HSP70 client protein

**DOI:** 10.4103/NRR.NRR-D-25-01872

**Published:** 2026-01-27

**Authors:** Joseph M. Irvin, Gail V.W. Johnson

**Affiliations:** Department of Biology, University of Rochester, Rochester, NY, USA; Department of Anesthesiology and Perioperative Medicine, University of Rochester, Rochester, NY, USA

**Figure 1:**
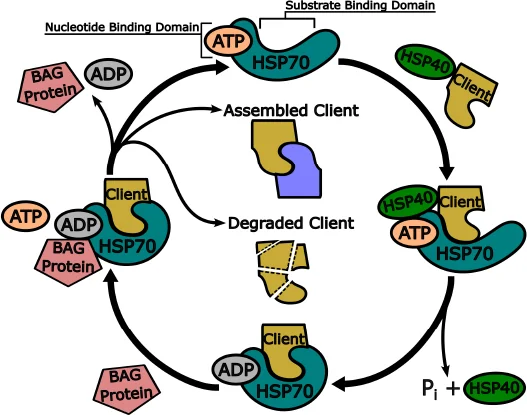
The BAG:HSP70 chaperone system. HSP70 chaperone behavior relies on a central ATP-dependent conformational shift. ATP-bound HSP70 is open and accessible for HSP40-assisted client capture. Subsequent ATP hydrolysis facilitated by HSP40 results in lid closing and a tight clamp down onto the substrate. A single BAG family protein will associate with the nucleotide binding domain and perform an ADP to ATP nucleotide exchange that reopens HSP70 and facilitates client release. The multi-domain nature of BAG proteins heavily influences when and where client release occurs, ultimately deciding whether the client achieves its native state or becomes degraded by autophagy or the proteasome. Created with Inkscape. ADP: Adenosine diphosphate; ATP: adenosine triphosphate; BAG: Bcl-2-associated athanogene; HSP: heat shock protein; Pi: phosphate.

Following translation, newly synthesized proteins must navigate a turbulent free energy landscape that threatens to trap them in misfolded or non-functional conformation. To reach their native states, many proteins are assisted by molecular chaperones that transiently facilitate folding and assembly without being part of the final structure. One such molecular scaffold is the heat shock protein 70 (HSP70) chaperone family, which acts in concert with Bcl-2-associated athanogene (BAG) co-chaperones to regulate a wide array of clients (**Figure 1**). By directly binding both a BAG protein and a client substrate, HSP70 acts as a versatile platform able to identify, retain, and effectively neutralize misfolded or mislocalized proteins. BAG proteins act as the decision-makers in this system, and modular interchange of the BAG family members BAG1–BAG6 influences client retention, localization, and degrative fate depending on the BAG protein bound. In a previous review discussing the diverse roles of BAG family proteins in mediating central nervous system (CNS) homeostasis, we performed an interactome analysis using publicly available datasets for all BAG family proteins except for BAG4, due to its limited expression in CNS contexts (Lin et al., 2025). In addition to expected canonical associations with HSP70 family members and the E3 ligase C-terminus of Hsc70-interacting protein, we were surprised to observe methyltransferase-like 3 (METTL3) as a common interactor of all BAG proteins examined.

METTL3 is the catalytic subunit of a nuclear methyltransferase complex that performs the essential task of installing N6-methyladenine (m^6^A) onto nascent mRNA (Su et al., 2023). It was therefore unexpected for this protein to be observed as a common interactor of the predominantly cytosolic BAG family proteins. While noncanonical cytoplasmic functions of monomeric METTL3 have been described mainly in cancer contexts, physiological METTL3 functions almost exclusively in the nucleus as a writer of m^6^A and stabilizer of nucleoli, with critical CNS effects on synaptic plasticity, calcium functioning, and consolidation of long-term memory (Su et al., 2023). In this perspective, we propose that chaperone control of METTL3 is likely a mandatory step in ensuring physiological functioning of the protein, with preliminary evidence to support METTL3 as a classic BAG:HSP70 client protein. BAG family proteins modularly bind HSP70 to drive client outcomes governing complex assembly, localization, and degradative fate. We hypothesize that this regulatory effect extends to METTL3, with BAG:HSP70 complexes acting as a versatile regulatory platform enabling proper METTL3 function in the CNS.

METTL3 in the cell exists within two different functional modes. The first and most well described mode of METTL3 involves nuclear functions of this enzyme in complex with methyltransferase-like 14 (METTL14), which together perform the critical physiological task of installing m6A modifications onto mRNA. The second and more recently discovered METTL3 mode involves its cytoplasmic role as a monomeric translation-enhancement scaffolding protein, which so far has been described only in the context of cancer biology (Choe et al., 2018). We propose that the BAG:HSP70 chaperone system may be a central regulatory guardrail for METTL3, capable of facilitating its physiological nuclear mode while also obstructing its pathological cytoplasmic mode. We begin by reviewing these modes of METTL3 function in a CNS context and conclude with a discussion of evidence and implications for METTL3 as a BAG:HSP70 client protein.

**METTL3 functional modes:** In its central canonical role, METTL3 is the catalytic subunit of a writer complex that installs m^6^A onto nascent mRNA transcripts in a sequence specific manner. m^6^A methylation is one of the most common epigenetic modifications of mRNA and is interpreted by reader proteins that influence transport, stability, splicing, and translation of modified transcripts. Neurons in particular rely heavily on post-transcriptional control to ensure effective translation in distal dendrites far from the soma. Beyond its central contributions to the mRNA epitranscriptome, the METTL3:METTL14 complex has several emerging roles in other areas of nuclear regulation. It has been recently demonstrated that the METTL3:METTL14 complex is capable of targeting DNA for installation of m^6^A marks, which act as protective features that support genome stability (Conti et al., 2025). Additionally, the METTL3:METTL14 complex has been shown to stabilize nucleoli by mediating degradation of specific histone methylases that would otherwise disrupt nucleolar liquid-liquid phase separation (Shan et al., 2024). Taken together, METTL3 is a key enzyme governing mRNA transcript fate, with additional contributions to genome stability and nuclear organization. When METTL3 functions properly, it has been demonstrated to support CNS processes such as neurogenesis, synaptic plasticity, neurodevelopment, and memory formation. However, the dysfunction and mislocalization of METTL3 have been associated with cancers, Alzheimer’s disease, traumatic brain injuries, and major depressive disorder (Lv et al., 2023).

In its more recently discovered cytoplasmic role, METTL3 acts independently of METTL14 to enhance translation of m6A labeled transcripts. Cytoplasmic METTL3 repurposed from its physiological role as a nuclear m^6^A writer takes on an m^6^A reader-like character. As a monomer, METTL3 will recognize m^6^A near stop codons and recruit translation initiation factors that increase ribosomal entry and enhance translation. In this way, it is speculated that cancer cells utilize m^6^A labeled transcripts to amplify oncoproteins favoring growth and survival, with proven METTL3 effects on cell proliferation, migration, apoptosis, glycolysis, and oncoprotein translation (Esteva-Socias and Aguilo, 2024). This translation-enhancement functionality is performed by METTL3 as a monomer, entirely independent from its catalytic methyltransferase activity. While these observations do not entirely discount this translation enhancement effect as a physiological occurrence, levels of non-nuclear METTL3 increase dramatically in pathological contexts, which suggests the cytoplasmic presence of this protein may be a mislocalization event hastening pathology development and oncogenesis.

**Figure 2:**
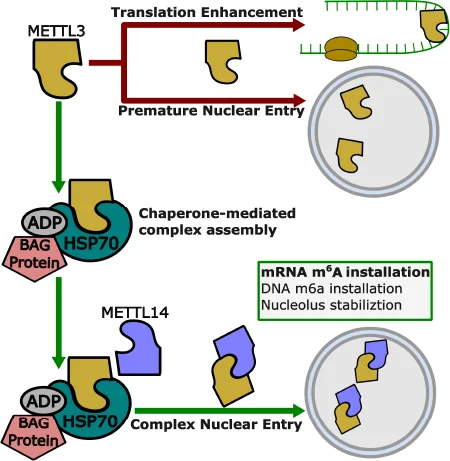
Chaperone-assisted METTL3 complex assembly. Following translation, cytoplasmic METTL3 has the immediate ability to enhance translation or enter the nucleus as a monomer, both pathological behaviors associated with cancers. Recognition and binding by the BAG:HSP70 chaperone complex is proposed to hold METTL3 in an inactive conformation until association with its partner METTL14. Formation of the canonical methyltransferase complex would then induce an allosteric change in METTL3 and release the full methyltransferase complex for nuclear entry and subsequent physiological functioning. Created with Inkscape. ADP: Adenosine diphosphate; BAG: Bcl-2-associated athanogene; HSP70: heat shock protein 70; m^6^A: N6-methyladenine; METTL3: methyltransferase-like 3.

**METTL3 as a BAG:HSP70 client protein:** Before speculating about regulatory control of this system, it is crucial to understand the transport and behavior of both METTL3 and METTL14. METTL3 has a verified nuclear localization sequence and may freely transition into the nucleus as a monomer. However, METTL14 lacks a nuclear localization sequence and is entirely reliant on the presence of METTL3 for nuclear translocation (Schöller et al., 2018). This relationship indicates that METTL3 and METTL14 must assemble into complex in the cytoplasm before crossing the nuclear membrane as one functional unit. The cell is presented with a problem: though METTL3 is capable of nuclear entry as a monomer, no studies have indicated METTL3 has relevant nuclear roles independent of METTL14. Therefore, METTL3 and METTL14 must be imported into the nucleus at a nearly 1:1 stoichiometric ratio with tight controls in place to prevent premature entry of the METTL3 subunit. We propose that this missing regulatory link fits the profile of established chaperone pathways and hypothesize that the BAG:HSP70 chaperone system acts to bind, stabilize, and sequester monomeric METTL3, thereby enabling complex assembly and preventing premature nuclear import (**Figure 2**).

The need to finish complex assembly prior to entering the nucleus situates METTL3 as a classic example of a protein that would be subject to chaperone control. In our proposed regulatory framework, newly translated cytoplasmic METTL3 is quickly recognized and stabilized by the binding of HSP70, with eventual client fate determined by the subsequent association of different BAG family co-chaperones. The immediate effect of METTL3 association with BAG:HSP70 would be twofold: cytoplasmic retention by masking of the nuclear localization sequence and a strong inhibition of METTL3 translation-enhancement. It has long been established that client proteins held by chaperones are kept in an inactive conformation that blocks recognition sites for unwanted localization and catalysis (Mayer and Bukau, 2005). BAG proteins that encourage long-term client retention, such as BAG3 and BAG6, are particularly important in this process and promote HSP70 holdase behavior. The longer METTL3 is inactive and stabilized in the cytoplasm, the more chance it has to recognize and bind its partner METTL14. This pattern fits the mold of other BAG:HSP70 client subunits in need of stabilization pending complex assembly. An example of a similar system is the BAG3:HSP70 retention of one subunit of the heterodimer CapZ, which is captured and held by BAG3 and HSP70 until becoming integrated into its functional complex (Hishiya et al., 2010). Should complex assembly not occur, excess METTL3 held within the BAG:HSP70 complex is also primed for clearance. BAG1 promotes ubiquitination and proteasomal degradation of clients, while BAG3 is capable of promoting localization to the phagophore and clearance by autophagy (Minoia et al., 2014). In a complementary fashion, the binding of BAG2 decreases premature client degradation by displacing an attached E3 ubiquitin ligase until a folding or degradation decision is made (Carrettiero et al., 2022). Taken together, BAG co-chaperones enable HSP70 to be highly responsive in governing client fate, and METTL3 is a prime example of a protein that would require this kind of regulation. Our observation that METTL3 and members of the BAG family interact corroborates this assumption and indicates that METTL3 may be sequestered as part of a BAG:HSP70 chaperone complex prior to assembly with METTL14 and physiological functioning. The model that METTL3 might be held by chaperones prior to METTL14 binding and nuclear import is not speculative as a mechanism class. Indeed, the importance of METTL3 in both physiological processes and disease emphasizes the need for such regulatory guardrails. By retaining inactive METTL3 in the cytoplasm as a chaperone client, the cell may temporally and spatially attenuate its activity pending complex assembly. The most likely candidate to fill this role is the BAG:HSP70 chaperone system, which would act as a cytoplasmic sink supporting assembly of the protein complex for neuro-physiological nuclear functions while also reducing availability of the protein monomer for pathologic cytoplasmic functions. Identification of METTL3 as a BAG:HSP70 client protein is an important development that would open the door to a new class of regulatory control and therapeutic development for this protein. This speculative interaction therefore warrants experimental validation in both pathological and physiological contexts.

*This work was supported by NIH grant, No. R01AG073121 (to GVWJ).*
